# Comparison of blood pressure measurements between the Huawei Watch D smartwatch application and the validated Omron M3 Intellisense device: Observational study

**DOI:** 10.1113/EP093286

**Published:** 2025-12-26

**Authors:** Jorge Velázquez Saornil, Raúl Frutos Llanes, Sonia Jiménez Blanco, Zacarías Sánchez Milá, José Manuel Barragán Casas, Rosario Pastor Martín, Angélica Campón Chekroun, David Rodríguez Sanz

**Affiliations:** ^1^ Facultad de Ciencias de la Salud. Departamento de Fisioterapia. Universidad Pontificia de Salamanca Salamanca Spain; ^2^ NEUMUSK Group, Facultad de Ciencias de la Salud, Departamento de Fisioterapia Universidad Católica de Ávila Ávila Spain; ^3^ Departamento de Fisioterapia. Aquacheck, Physiotherapy clinic GESMUTE member Cáceres Spain; ^4^ Facultad de Enfermería, Fisioterapia y Podología Universidad Complutense de Madrid Madrid Spain

**Keywords:** blood pressure, precision health, precision medicine, smartwatch, validation

## Abstract

Popular wearable devices that record a variety of health metrics, such as real‐time blood pressure (BP), could play a role in detecting hypertension in the population. The objective of this study was to compare the validity of the Huawei D device with that of the Omron M3 Intellisense device used by health workers. Sampling was used to obtain patients with normal and abnormal BP. Measurements were taken on the wrist via the Huawei Watch D, while measurements were made using the Omron M3 Intellisense (prevalidated) device used by experienced healthcare personnel at the health centre. A total of 100 normotensive and 100 hypertensive patients were analysed. The concordance statistics were calculated, and linear regression analysis was performed. The intraclass correlation coefficient was calculated to assess the concordance between the instruments used in the measurement and among the observers who made the measurements under a two‐factor mixed‐effects model, with calculation of the degree of absolute concordance in mean measurements. We found moderate results (systolic blood pressure (SBP) at 30 and 60 min and heart rate at 30 min) and nil for the rest [0.63 (0.36–0.78) < 0.001]; in addition, the comparison of the two groups was verified by a Bland–Altamn graph, in which the disparity of the results was observed. The Huawei D BP measuring device was shown to be relatively effective for measuring BP in hypertensive patients. However, the specificity of BP measurement in normotensive patients was not as reliable as in hypertensive patients.

## Abstract (highlights)

- **What is the central question of this study?**
  How does automatic blood pressure measurement by a new smartwatch compare with blood pressure measurementby an experienced healthcare professional?
- **What is the main finding and its significance?**
  Blood pressure measurements taken by the smartwatch device are not highly reliable in comparison to measurements taken by a healthcare professional.

## 1 INTRODUCTION

Cardiovascular disease (CVD) is the leading cause of death worldwide, with 17.9 million deaths each year. The Global Burden of Cardiovascular Diseases and Risk Factors study (Booth, 1977; Roth et al., 2020; Silberschmidt, 2021) revealed hypertension to be the most prevalent CVD (65.5%) in 2019, followed by ischaemic heart disease (11.4%), peripheral vascular disease (6.5%), cerebrovascular disease or stroke (5.8%), arrhythmia (atrial fibrillation and atrial flutter, 3.5%), rheumatic heart disease (2.3%), heart failure (1.5%), coronary heart disease (1.1%), hypertensive heart disease or cardiopathy (1.08%), congenital heart disease (0.7%) and cardiomyopathies (0.53%) (Mazoteras et al., 2019; Stergiou et al., 2022). These results demonstrate how crucial it is to underpin efforts for the prevention and treatment of hypertension to reduce the global incidence of CVD (Ordunez et al., 2024).

CVD affects organs such as the heart and blood vessels, which can lead to stroke, coronary heart disease and rheumatic diseases. More than 80% of CVD‐related deaths are attributable to coronary heart disease and stroke, and 33% of these deaths occur prematurely, in people <70 years of age (Takahashi et al., 2013). The prevention of premature CVD‐related deaths requires identification of those at high risk and ensuring that they receive appropriate treatment. In this context, access to essential medicines and basic health technologies for the recording and treatment of such non‐communicable diseases in all primary care settings is essential to provide treatment and counselling to affected individuals (Silberschmidt, 2021; Stergiou et al., 2022).

High blood pressure (BP) is defined as a BP of 140/90 mmHg or higher in adults >18 years of age who are not taking medication for hypertension (which might increase the incidence of adverse cardiovascular events in the obese population; Booth, 1977; Kikuya et al., 2002; Montoye et al., 2016; Steeves et al., 2011; Tryon & Williams, 1996; Verberk et al., 2007). The prevalence of this disease worldwide is ∼20%–30%. These events are recognized by clinicians and health policy‐makers as significant health problems because of their various secondary impacts on morbidity, mortality, and medical and economic costs (Han et al., 2023; Piper et al., 2014).

The sphygmomanometer, also known as a BP gauge, is a device used to measure blood pressure. It is composed of an inflatable cuff that is used to collapse and then release the artery under the cuff in a controlled manner, with a mercury or aneroid manometer to measure the pressure. Manual sphygmomanometers are used with a stethoscope when the auscultatory technique is used (Steeves et al., 2011). This measurement should be performed by a qualified healthcare provider with training and experience, in a medical centre. Currently, BP monitoring is being performed with new technologies, including mobile applications, to obtain even greater benefits than can be obtained with conventional devices (Ogedegbe & Pickering, 2010; Roth et al., 2020).

Although standard validation protocols exist, many devices on the market have not been tested for accuracy. These devices can record BP from the upper arm, wrist or finger, but the upper arm is preferred (Booth, 1977). Twenty‐four‐hour ambulatory monitoring is the best predictor of cardiovascular risk in individual patients and is the only technique that can accurately describe the diurnal rhythm of blood pressure. Ambulatory monitoring is mainly used for the diagnosis of hypertension, whereas self‐monitoring is used to monitor the response to treatment (Anguita Sánchez, 2013; Rosenberg, 2019). Different BP measurement techniques may be preferred in certain situations (Holtermann et al., 2011). In infants, the ultrasound technique is best, but during pregnancy and after exercise, diastolic pressure can be difficult to measure with the conventional auscultatory method (Ogedegbe & Pickering, 2010). In obese subjects, it is important to use a cuff of the correct size (Piper et al., 2014).

For these reasons, the aim of the present study was to compare the validity of smartwatches (the Huawei Watch D smartwatch) used by patients for validated sphygmomanometry used by healthcare staff to assess normotensive and hypertensive subjects to obtain reliable BP measurements and thus reduce the number of visits to health professionals for this reason.

## 2 MATERIALS AND METHODS

### 2.1 Ethical approval

All patients received a study information sheet, and an informed consent form was signed before the start of the study. The research was conducted in accordance with the principles and recommendations of STROBE and conformed to the standards set by the latest revision of the *Declaration of Helsinki*. Biomedical research criteria were respected throughout the study. A record of the clinical trial was not made because it was an observational study and did not involve any intervention on patients, only the measurement of BP with two different devices. The study was approved by the Ethics Committee of the Nuestra Señora de Sonsoles Hospital Complex, with registration number GASAV/2023/05 for the development of this project.

This research study was carried out by a group of healthcare professionals composed of nurses and physicians with >15 years of experience in the care of hypertensive patients. The professionals who carried out the measurement were not blinded; however, the evaluators were blinded, hence the study is simply blind. The devices used in the investigation were as follows.

**FIGURE 1:**
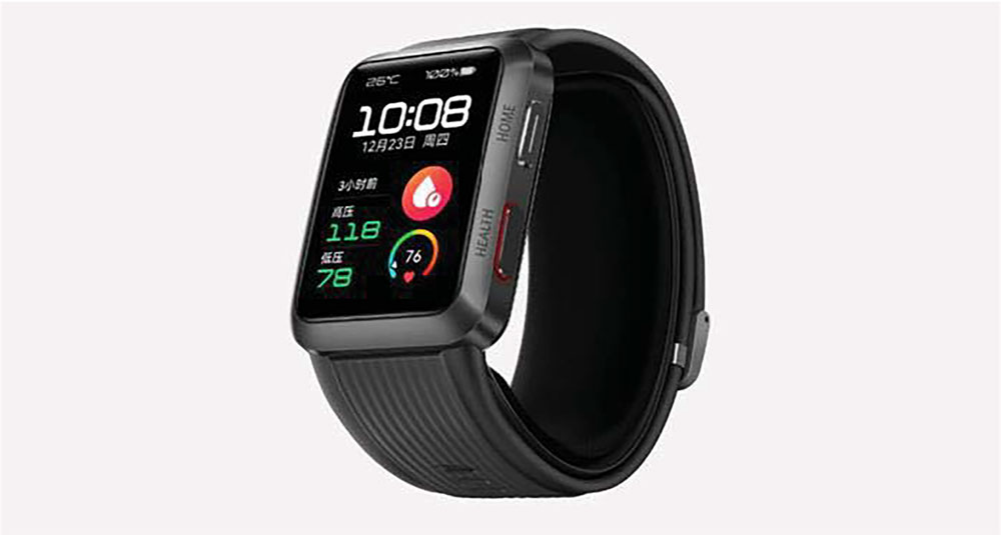
Huawei D device.

**FIGURE 2:**
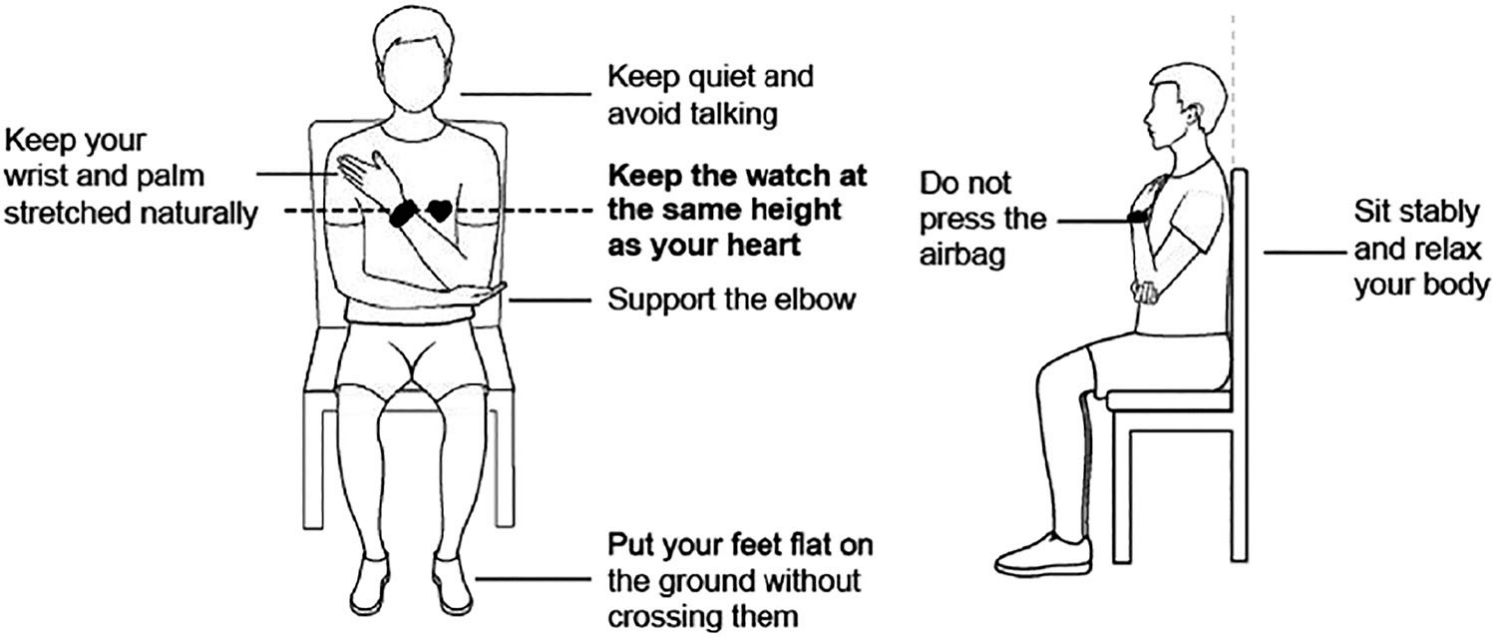
Device instructions.

1. The Omron M3 Intellisense (Omron Healthcare, Kyoto, Japan) was the standard device used as the reference point. The device was recently validated for the general population according to an international protocol (Takahashi et al., 2021). The Omron M3 Intellisense is an automated upper‐arm oscillometric device for the arm that measures systolic blood pressure. The standard upper arm cuff of the device is designed for upper arm circumferences of 22–32 cm, and a large upper arm cuff is also available for upper arm circumferences of 32–42 cm (Smith et al., 1985). Prior to this, the BP measurement with the Omrom 3 device was checked against the tension measurement with the standard reference, which was compared with the measurement blood pressure measurement using the standard reference, which was performed by a qualified professional using a sphygmomanometer. In addition, the device was validated by Akpolat et al. (2011).
2. The Huawei Watch D smartwatch (Huawei Technologies Co., Guangdong, China) is a device that can take BP measurements via pressurized air, and thus achieves the highest possible reliability of BP readings, and this device is registered in China as a medical device. The device used for the research is shown in Figure 1. For accurate BP measurements, the Huawei Watch D uses a mini‐pump housed in the strap that electrically inflates an air chamber to exert pressure on the arteries in the wrist and accurately measures blood pressure. It is capable of tracking both the systolic and diastolic pressure values, and the manufacturer claims the accuracy to be ±3 mm of mercury (Zhang et al., 2022). The manufacturer's instructions on position and posture were considered for accurate reading. The measurement method can be seen in Figure 2.

### 2.2 Patients and recruitment

In this study, there were two arms of participants: one group with manometric BP measurements and another group with BP recordings via a smartwatch. Participants who were scheduled for ambulatory BP monitoring were recruited by their primary care physician and fitted with a smartwatch (Huawei Watch D smartwatch) for BP measurement on their arm. After calibration, four measurements were performed: (1) initial time; (2) 30 min after the first recording; and (3) 60 min after the first recording. In addition, the BP of another group of patients was measured by a qualified expert (with >15 years of experience and >25 h of clinical activity per week) via manometry on an outpatient basis. This action was carried out in 100 normotensive and 100 prediagnosed hypertensive participants, considering the sample size calculation measured via the Granmo tool (Regicor, Barcelona, Spain).

The inclusion criteria were men and women who were >18 years of age. The inclusion criteria for subjects with hypertension was a history of hypertension diagnosed in medical history. The exclusion criteria were as follows: (1) sustained arrhythmia; (2) circulatory problems that contraindicated the use of the cuff; (3) pregnancy; (4) neurological history that might cause cardiovascular alterations; or (5) taking drugs that might cause cardiovascular alterations. An elimination criterion was if, during the measurement, any pharmacological treatment related to cardiac alterations were to be added.

The variables to be collected were height (quantitative variable, measured in metres, with a validated measuring rod), weight (quantitative variable, measured in kilograms with a validated scale), body mass index (quantitative variable, according to the Quetelet equation), gender (nominal dichotomous variable), BP (quantitative variable, measured in millimetres of mercury), heart rate (quantitative variable, measured as the number of beats per minute), arm circumference and wrist circumference (in centimetres), diabetes mellitus, dyslipidaemia, smoking, drinking, heart disease, kidney disease, CVD and hypertension treatment.

The same measurements were carried out in both groups (100 hypertensive and 100 normotensive subjects) and with the two devices described above that measure BP. During the measurement, for both groups, the individual remained quiet, still, seated with a straight back and without moving, keeping their feet on the floor in a parallel position, without crossing their legs (Noh et al., 2024). They rested their arms on a flat surface, with the palm of the hand facing upwards and the elbow slightly bent such that the arm was at the level of the heart. The interval between BP measurements was 30–60 min. All the measurements were carried out btween 09:00 and 10:00 h, in the same room and by the same healthcare professional.

With respect to the statistical analysis, a normality analysis was carried out via the Shapiro–Wilk test. Descriptive statistics were performed, with the inclusion of measures of central tendency and their ranges of dispersion for quantitative variables and with frequency and percentage for qualitative variables. BP variability was calculated via the mean true variability, SD and coefficient of variation (Lee et al., 2021, 2023). The Bland–Altman method and Taffé method were used for the assessment of bias and precision. SPSS 22.0 software (IBM SPSS Statistics for Windows, IBM Corp., Armonk, NY, USA) was used.

## 3 RESULTS

A total of 200 participants were examined (100 subjects in the hypertensive group and 100 subjects in the normotensive group). Table 1 shows the descriptive variables of the sample.

**TABLE 1:** Descriptive sample.

Charactersitic	Hypertensive	Normotensive
Age, years, mean (SD)	69.06 (12.24)	21.03 (1.61)
Sex, *n*		
Male	45	39
Female	55	61
Wrist circumference, *DT*	1.57	17.19 (1.51)
Body mass index, kg m^−2^, mean (SD)	29.49 (6.04)	23.48 (2.58)
Diabetes mellitus, *n*		
No	72	100
Yes	28	0
Dyslipidaemia, *n*		
No	32	100
Yes	68	0
Smoker, *n*		
No	94	44
Yes	6	25
Drinker, *n*		
No	96	29
Yes	4	40
Cardiac pathology, *n*		
No	73	100
Yes	27	
Nephropathy, *n*		
No	98	100
Yes	2	
Cardiovascular diseases, *n*		
No	94	100
Yes	6	
Antihypertensive treatment, *n*		
No	12	
Yes	88	

### 3.1 Hypertensive subjects

The descriptive values of BP and heart rate for each of the devices used in hypertensive patients can be seen in Table 2.

**TABLE 2:** Descriptive values of blood pressure and heart rate according to device used in hypertensive patients.

	Huawei		Omron	
Parameter	Range	Mean (SD)	Range	Mean (SD)
Systolic blood pressure, mmHg				
0 min	98–172	137.86 (15.15)	99–195	139.37 (17.37)
30 min	98–176	134.61 (17.18)	95–191	133.85 (17.01)
60 min	102–177	134.29 (16.71)	97–183	135.23 (17.59)
Diastolic blood pressure, mmHg				
0 min	43–100	78.33 (9.28)	56–108	80.97 (10.08)
30 min	40–106	77.45 (11.19)	55–109	79.27 (10.45)
60 min	44–101	77.69 (10.25)	57–106	79.67 (10.00)
Heart rate, beats min^−1^				
0 min	45–113	76.11 (14.21)	44–110	74.99 (12.87)
30 min	48–115	73.64 (13.49)	49–119	73.66 (12.88)
60 min	48–110	74.30 (14.10)	46–115	72.41 (12.97)

To quantify the reliability (intra‐ and inter‐apparatus) of the measurements associated with the quantitative variables, the intraclass correlation coefficient (ICC) was calculated to assess the agreement between the instruments used in the measurement and between the observers who made the measurements under a two‐factor mixed‐effects model, in which the effects of the persons are random and the effects of the measurements are fixed (Meyer et al., 2020), calculating the degree of absolute agreement in the average measurements. ICC values of <0.4 represent low reliability, values between 0.4 and 0.75 represent fair to good reliability, and values of >0.75 represent excellent reliability (Falter et al., 2022). The degree of agreement achieved for each of the variables measured was excellent for each of the devices used and can be seen in Table 3. The degree of agreement achieved for each of the variables between devices was excellent and can be seen in Table 4.

**TABLE 3:** Reliability of intradevice blood pressure and heart rate measurements in hypertensive patients.

	Reliability		Force agreement
Parameter	ICC (95% CI)	*P*‐value	
Omron			
SBP	0.93 (0.89–0.95)	<0.001	Very good
DBP	0.95 (0.93–0.97)	<0.001	Very good
HR	0.96 (0.95–0.97)	<0.001	Very good
Huawei			
SBP	0.90 (0.87–0.93)	<0.001	Very good
DBP	0.88 (0.83–0.91)	<0.001	Very good
HR	0.95 (0.93–0.96)	<0.001	Very good

Abbreviations: CI, confidence interval; DBP, diastolic blood pressure; HR, heart rate; ICC, intraclass correlation coefficient; SBP, systolic blood pressure.

**TABLE 4:** Reliability of inter‐apparatus blood pressure and heart rate measurements in hypertensive patients.

	Reliability		Force agreement
Parameter	ICC (95% CI)	*P*‐value	
Systolic blood pressure			
0 min	0.87 (0.80–0.91)	<0.001	Very good
30 min	0.76 (0.64–0.84)	<0.001	Very good
60 min	0.96 (0.94–0.97)	<0.001	Very good
Diastolic blood pressure			
0 min	0.91 (0.86–0.94)	<0.001	Very good
30 min	0.77 (0.66–0.85)	<0.001	Very good
60 min	0.95 (0.93–0.97)	<0.001	Very good
Heart rate			
0 min	0.91 (0.86–0.94)	<0.001	Very good
30 min	0.79 (0.68–0.86)	<0.001	Very good
60 min	0.96 (0.93–0.98)	<0.001	Very good

Abbreviations: CI, confidence interval; ICC, intraclass correlation coefficient.

### 3.2 Normotensive subjects

The results of the normotensive patients included in the study were as follows. Table 5 shows the descriptive values of BP and heart rate in each of the devices in normotensive patients. Table 6 shows that the degree of agreement achieved for each of the variables was very low for each of the devices used in normotensive patients.

**TABLE 5:** Descriptive values of blood pressure and heart rate according to devices in normotensive patients.

	Huawei		Omron	
Parameter	Range	Mean (SD)	Range	Mean (SD)
Systolic blood pressure, mmHg				
0 min	99–136	119.32 (9.18)	76–135	113.60 (11.11)
30 min	111–136	123.24 (7.21)	78–136	114.68 (13.54)
60 min	62–127	96.93 (18.84)	75–136	106.86 (18.20)
Diastolic blood pressure, mmHg				
0 min	69–119	78.39 (6.56)	69–87	76.86 (5.25)
30 min	69–92	77.78 (5.13)	69–87	77.88 (5.60)
60 min	69–87	77.66 (5.58)	69–87	78.30 (5.72)
Heart rate, beats min^−1^				
0 min	60–98	81.05 (10.39)	62–98	79.63 (10.30)
30 min	62–98	79.42 (10.95)	62–97	80.34 (10.59)
60 min	62–98	80.12 (10.86)	62–98	81.23 (10.16)

**TABLE 6:** Reliability of intradevice blood pressure and heart rate measurements in normotensive individuals.

	Reliability		Force agreement
Parameter	ICC (95% CI)	*P*‐value	
Omron			
SBP	−0.01 (−0.38 to 0.27)	0.523	Low
DBP	−0.12 (−0.56 to 0.21)	0.741	Low
HR	−0.13 (−0.59 to 0.20)	0.755	Low
Huawei			
SBP	0.12 (−0.07 to 0.30)	0.05	Low
DBP	0.04 (−0.35 to 0.32)	0.412	Low
HR	0.06 (−0.31 to 0.34)	0.352	Low

Abbreviations: CI, confidence interval; DBP, diastolic blood pressure; HR, heart rate; ICC, intraclass correlation coefficient; SBP, systolic blood pressure.

Table 7 shows that the degree of agreement achieved in each of the inter‐apparatus variables was different according to the time at which it was measured. Thus, we obtained moderate results (systolic blood pressure (SBP) at 30 and 60 min and heart rate at 30 min) and were null for the rest.

**TABLE 7:** Reliability of blood pressure and inter‐apparatus heart rate measurements in normotensive individuals.

	Reliability		Force agreement
Parameter	ICC (95% CI)	*P*‐value	
Systolic blood pressure			
0 min	0.60 (0.31–0.75)	< 0.001	Moderate
30 min	0.08 (−0.35 to 0.38)	0.337	Low
60 min	0.05 (−0.41 to 0.36)	0.393	Low
Diastolic blood pressure			
0 min	0.13 (−0.18 to 0.37)	0.192	Low
30 min	0.05 (−0.42 to 0.36)	0.397	Low
60 min	−0.09 (−0.63 to 0.27)	0.668	Low
Heart rate			
0 min	0.63 (0.36–0.78)	< 0.001	Moderate
30 min	−0.12 (−0.67 to 0.25)	0.709	Low
60 min	0.08 (−0.36 to 0.38)	0.332	Low

Abbreviations: CI, confidence interval; ICC, confidence interval.

**FIGURE 3:**
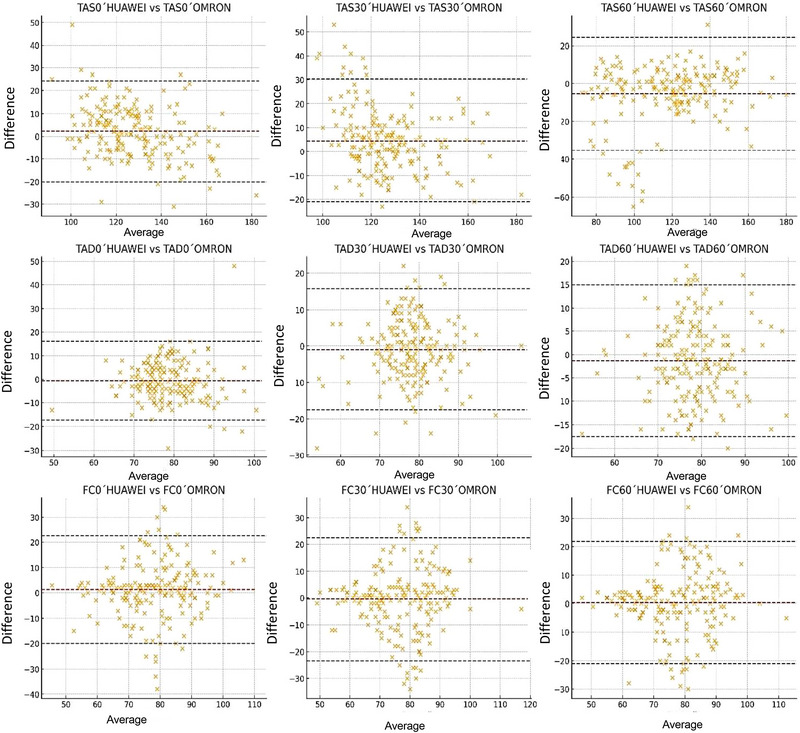
Intergroup Bland‒Altman graph.

The Bland–Altman graph (Figure 3) shows the agreement between the two groups at different times of BP measurement. In the Bland–Altman plots, a systematic discrepancy was evident between measurements obtained with the Huawei and Omron devices for systolic blood pressure, diastolic blood pressure and heart rate variables at different assessment times (rest, 30 and 60 min). In particular, the systolic blood pressure measurements showed a consistent negative bias, indicating that the Huawei smartwatch tends to underestimate values relative to the reference standard. This discrepancy was accentuated in the 60 min measurements, where wide limits of agreement were also observed, compromising the interchangeability between methods. In the case of diastolic blood pressure, although the differences were smaller, non‐negligible variability between devices persisted. For heart rate measurements, agreement was relatively better, with biases close to zero, although with significant dispersion in some cases, especially at 30 min. These findings suggest that although consumer devices. such as the Huawei device, might provide rough estimates, their use in clinical settings should be approached with caution, especially when diagnostic accuracy or rigorous therapeutic monitoring by highly qualified and experienced healthcare professionals is required.

## 4 DISCUSSION

This research is considered the first study to investigate the validation of a smart device, the Huawei Watch D, connected to an app to measure heart rate and BP in patients with hypertension. The results show that BP monitoring by hypertensive patients with the Huawei Smartwatch device is not highly reliable. The reliability of automatic BP devices can differ depending on the patient's condition (Falter et al., 2022). In hypertensive patients, elevated BP makes it easier to detect the point of maximum oscillation in oscillometric cuffs, which might improve accuracy (Kuwabara et al., 2020). However, in normotensive subjects, where BP is at lower levels and with greater variability, the devices might be less accurate. This might lead to more consistent measurements hypertensive patients and fewer fluctuations than in normotensive patients, whose BP might vary more widely because of greater arterial compliance and the influence of the autonomic nervous system (Lee et al., 2021; Piper et al., 2014).

Traditionally, people check their BP by going to their local health centres, but the advent of smart devices in our lives, mainly mobile phones, has been revolutionary (Lessof et al., 2023). In 2019, a survey of 27 countries by the Pew Research Centre revealed that >2.5 billion people used smartphones, with South Korea having the highest usage rate, with 95% of its population owning one (Silberschmidt, 2021).

Based on these smartphones and the use of sensors and software applications, BP can be monitored through wearable smartwatches via photoplethysmographic or oscillometric technology. Thus, individuals can measure BP by themselves, which has been shown to increase self‐monitoring, awareness, prognosis and treatment adherence in hypertensive patients, including the possibility of taking several measurements in a single day and even continuous measurements (Han et al., 2023).

Globally, the use of home BP monitoring is increasing in several countries, because it is a useful adjunct to clinical measurements, with significant acceptance by patients with hypertension and several advantages (Imai et al., 2007; Montoye et al., 2016). Patients with hypertension can use a validated sphygmomanometer at home, which appears to be particularly cost‐effective (Akpolat et al., 2011; Asmar et al., 2010; Wang et al., 2022).

Self‐measurement of BP at home is becoming an increasingly popular practice among people of all ages and is even recognized by healthcare professionals as an aid to self‐management and good therapeutic monitoring of BP. Its ease of measurement, together with research support for practice, makes it a mainstay in the diagnosis of hypertension (John & Freedson, 2012). However, current 24 h ambulatory BP monitoring devices must be worn throughout the day with a cuff around the arm and connected to a recording unit hanging at waist level, which is uncomfortable to wear and limits the natural movements of the patient; thus, the portable wrist devices are gradually gaining importance and acceptance by both professionals and the general population. One of the minor drawbacks of these watches is that, in order to measure BP adequately, the patient must stop whatever activity he or she is doing, in order to sit or stand and place the watch hand at the level of the heart for a more accurate reading, because some studies have shown that variations of ∼10 cm from this height change BP by ∼7 mmHg (Kuwabara et al., 2020). Therefore, an obvious limitation results from the inability to monitor BP continuously throughout the day and even during sleep.

The main disadvantage of automatic sphygmomanometers for home use is their inaccuracy, although their accuracy is progressively improving (Kikuya et al., 2002). This inaccuracy is more prevalent in disease‐specific populations, which might require additional validation (Imai et al., 2007; Mazoteras‐Pardo et al., 2020). The 2010 European Society of Hypertension Practice Guidelines for home BP monitoring recommended specific validation tests for patients with end‐stage chronic kidney disease (Montoye et al., 2016). Arterial stiffness can influence the correspondence between readings measured with mercury and oscillometric devices (Troiano et al., 2008; Yi et al., 2022). In contrast, Ahn et al. (2021) validated BP measurement in patients with Parkinson's disease via a smartwatch and demonstrated that was reliable and accurate despite the inherent resting tremors of these patients.

This validation has been conducted following the European Society of Hypertension guidelines, although a validated non‐invasive oscillometric device (Omron M3 Intellisense) for the upper arm has been used as a reference instead of a mercury sphygmomanometer (McGraw & Wong, 1996). Our watch is compatible with oscillometry and photoplethysmography technologies and detects blood pressures between 0 and 300 mmHg and heart rates from 40 to 180 beats min^−1^. In addition, all these data can be stored and analysed for later reference by installing the Huawei Health app on a smartphone (Zhang et al., 2022).

Yi et al. (2022), studying the effectiveness of this watch in measuring resting BP, monitoring 24 h systolic blood pressure, alerting to diurnal BP spikes and ambulatory BP measurement, demonstrated that the Huawei D could detect abnormal BP rhythms and hypertension spikes and that resting BP and ambulatory measurements were useful in the early diagnosis of the disease. Like other studies, they also demonstrated its efficacy in increasing awareness and self‐management of hypertension among patients (Noh et al., 2024).

Recently, smartwatches have been designed to meet the medical requirements proposed by the International Organization for Standardization (ISO 81060‐2:2018) for non‐invasive sphygmomanometers, but recently new implementation standards have been postulated (Fleiss, 2011). Our watch, the Huawei Watch D, complies with criteria 1 and 2 of the Association for the Advancement of Medical Instrumentation/European Society of Hypertension/International Organization for Standardization (AAMI/ESH/ISO) universal standard guidelines (ISO 81060‐2:2018) (Lee et al., 2023; Zhang et al., 2022), which shows that it could be a reliable device for daily self‐monitoring of BP. The findings of the present study showed that the Huawei D device successfully passed the validation requirements for CVD patients (John & Freedson, 2012). However, our findings might not be generalizable to other specific populations with specific diseases, such as elderly or diabetic patients, in addition to pregnant women, because these conditions have not been addressed. Furthermore, CVD patients can present with a specific type of CVD, and future studies should be conducted to develop further validations of applications according to specific recommendations in patients with each type of CVD (Wan et al., 2010). However, standard validation protocols do not require these measures. Finally, consecutive sampling bias should be considered in the present study, and a simple random sampling process might be more appropriate for future studies.

The Omron HEM‐6410T‐ZM and Omron HEM‐6410T‐ZL watches were the first wearable devices capable of measuring BP, and both also met the validation criteria of ANSI/AAMI/ISO 81060‐2:2018 (Akpolat et al., 2011; Saito et al., 2019; Wang et al., 2022). In comparison, the Huawei Watch D is a smaller watch, with a wrist cuff circumference range of 13–20 cm, which is more adaptable than Omron devices, measuring 16–21.5 cm, making it more accessible for smaller wrists of thin people. In our case, the mean wrist circumference was 17.35 cm, hence there was no impediment to assessing BP in our participants.

There are two major position papers on the use of smartwatches for BP measurement. One study was conducted in 2021 by the Korean Society of Hypertension (Lee et al., 2021), and the other in Europe in 2022 by the European Society of Hypertension Working Group (Takahashi et al., 2021). They argued that these watches are already a real alternative for BP measurement, raising awareness of the importance of BP monitoring and providing data on their variability and accuracy at home, but it is doubtful that they can be used to monitor the response to antihypertensive treatment in hypertensive patients and those with cardiovascular conditions.

For people suffering from hypertension, reliable BP measurement is essential for a correct diagnosis, treatment and better prognosis of the disease, to reduce the risk of CVD and even death (Lee et al., 2023). Today, measurements in healthcare settings are based on aneroid and electronic sphygmomanometers. Aneroid devices require the use of a stethoscope and are therefore not suitable for home use by the general population. Therefore, in these situations, electronic devices have become the best available option, and studies have suggested that self‐measurement of BP improves prognosis and reduces the need for antihypertensive treatment to a greater extent than measurement taken only in healthcare settings (Wan et al., 2010). Patients’ self‐awareness of their BP might be the key to improving disease awareness and self‐management of lifestyle changes and medical treatment (Mazoteras‐Pardo et al., 2019).

Finally, it is necessary to perform a cost‐effectiveness analysis of these smartwatches when measuring BP, because the inappropriate use of these devices can generate emotional and physical stress, false diagnoses and therapeutic errors, among other problems, which can lead to an increase in resource use and healthcare spending. Furthermore, an unvalidated device cannot be used or prescribed for BP measurement, but if it is marketed and patients buy it, it is necessary for healthcare professionals to be up to date on its use and indications, in order to advise and treat the individual appropriately. Therefore, and in agreement with a similar article (Yi et al., 2022), further studies in hypertensive and normotensive populations are needed to increase the validity and reliability of smartwatches in BP measurement.

The most important limitation of our study is the limited sample size, because we believe that more measurements could be performed on these patients. Furthermore, another potential limitation is the age difference between the two groups and that the study of these data could alter these results. Another limitation is the specificity in normotensive patients, because BP can fluctuate owing to various factors, such as stress, physical activity, diet and measurement technique. This could lead to false positives in normotensive patients, reducing the specificity of the study. The normotensive group was obtained from a younger sample to assess more stable scores with fewer changes than the hypertensive group, where baseline scores are higher and changes are greater. Additionally, a slight constant or proportional bias in BP assessment against the reference standard must be taken into account, but an evaluation and pretesting of the devices used against the sphygmomanometer measurement was performed, and the validation of the reference device was taken into account (Akpolat et al., 2011).

Further technological development of these devices and consistent validation standards are needed to increase the number of investigations in a standardized way if these devices are to be applied routinely and safely, even replacing cuff BP monitors in the future. Given the above, further clinical trials with anthropometric data, medication intake and underlying comorbidities of participants, together with prospective follow‐up data, are needed to define and assess the characteristics of users who would benefit most from the use of a smartphone‐ or smartwatch‐based BP monitor. As a future line of research, we can compare devices of the same age with different conditions, such as hypertension, diabetes or hypercholesterolaemia, also taking into account the impact of confounding variables, such as stress, diet and physical activity.

## 5 CONCLUSION

Analysis of agreement between Huawei and Omron devices for BP (systolic and diastolic) and heart rate measurements in normotensive and hypertensive subjects shows high variability between the two methods.

Overall, the diagrams show a slight to moderate systematic bias in most comparisons, especially for systolic pressure measurements, where differences tend to increase with higher mean values. Furthermore, the limits of agreement for several of the variables exceed clinically acceptable thresholds, suggesting that the devices are not interchangeable in contexts where accuracy is crucial, such as in hypertension monitoring or individualized therapeutic decisions.

Of particular concern are some measurements of diastolic pressure and heart rate at 60 min, where the dispersion of values suggests lower reliability of the Huawei device versus the validated standard (Omron). This disparity might be attributable to technological differences in the detection algorithm, pulse synchronization or non‐standardized measurement conditions. Consequently, the use of the Huawei device as a diagnostic or clinical monitoring tool should be considered with caution, especially in vulnerable populations. Systematic validation of such mass‐market devices through robust comparative clinical studies is recommended prior to deployment in healthcare settings.

## AUTHOR CONTRIBUTIONS

Conceptualization: Jorge Velázquez Saornil and Rosario Pastor Martín. Methodology: José Manuel Barragán Casas, Raúl Frutos Llanes and David Rodríguez Sanz. Software: Angélica Campón Chekroun, Rosario Pastor Martín and Zacarías Sánchez Milá. Validation: Rosario Pastor Martín and Zacarías Sánchez Milá. Formal analysis: Sonia Jiménez Blanco, David Rodríguez Sanz and Jorge Velázquez Saornil. Investigation: Jorge Velázquez Saornil, Sonia Jiménez Blanco and José Manuel Barragán Casas. Resources: Zacarías Sánchez Milá. Data curation: Sonia Jiménez Blanco, Raúl Frutos Llanes, Zacarías Sánchez Milá and José Manuel Barragán Casas. Writing—original draft preparation: Jorge Velázquez Saornil and David Rodríguez Sanz. Writing—review and editing: José Manuel Barragán Casas, Angélica Campón Chekroun and Raúl Frutos Llanes. Visualization: Jorge Velázquez Saornil and Zacarías Sánchez Milá. Supervision: Rosario Pastor Martín and David Rodríguez Sanz. Project administration: Jorge Velázquez Saornil. Funding acquisition: David Rodríguez Sanz. All authors approved the final version of the manuscript and agree to be accountable for all aspects of the work in ensuring that questions related to the accuracy or integrity of any part of the work are appropriately investigated and resolved. All persons designated as authors qualify for authorship, and all those who qualify for authorship are listed.

## CONFLICT OF INTEREST

The authors declare that they have no conflicts of interest. The funders had no role in the design of the study; in the collection, analyses or interpretation of data; in the writing of the manuscript; or in the decision to publish the results.

## Data Availability

Article data are available upon request from the author.
